# Unfolded protein response in hepatitis C virus infection

**DOI:** 10.3389/fmicb.2014.00233

**Published:** 2014-05-20

**Authors:** Shiu-Wan Chan

**Affiliations:** Faculty of Life Sciences, The University of ManchesterManchester, UK

**Keywords:** hepatitis C virus, unfolded protein response, endoplasmic reticulum stress, hepacivirus, virus-host interaction

## Abstract

Hepatitis C virus (HCV) is a single-stranded, positive-sense RNA virus of clinical importance. The virus establishes a chronic infection and can progress from chronic hepatitis, steatosis to fibrosis, cirrhosis, and hepatocellular carcinoma (HCC). The mechanisms of viral persistence and pathogenesis are poorly understood. Recently the unfolded protein response (UPR), a cellular homeostatic response to endoplasmic reticulum (ER) stress, has emerged to be a major contributing factor in many human diseases. It is also evident that viruses interact with the host UPR in many different ways and the outcome could be pro-viral, anti-viral or pathogenic, depending on the particular type of infection. Here we present evidence for the elicitation of chronic ER stress in HCV infection. We analyze the UPR signaling pathways involved in HCV infection, the various levels of UPR regulation by different viral proteins and finally, we propose several mechanisms by which the virus provokes the UPR.

## Introduction

Hepatitis C virus (HCV) infection produces a clinically important disease affecting around 3% of the world population (Thomas, 2013). The disease usually manifests itself as chronic hepatitis, which can progress into fibrosis, cirrhosis and eventually into hepatocellular carcinoma (HCC). How the virus establishes persistence and causes diseases is still far from clear. Understanding how the virus interacts with the host is key to answer these questions. Recently the unfolded protein response (UPR), a host homeostatic response to endoplasmic reticulum (ER) stress, has emerged to be a novel mechanism involved in a number of human diseases including that caused by virus infections (Dimcheff et al., 2003; Favreau et al., 2009; Wang and Kaufman, 2012). UPR has also been frequently manipulated by a number of viruses to aid in infection and to attenuate anti-viral response (Isler et al., 2005; Smith et al., 2006; Yu et al., 2006; Liu et al., 2009; Jheng et al., 2010; Ambrose and Mackenzie, 2011; Pena and Harris, 2011; Burnett et al., 2012; Galindo et al., 2012; Qian et al., 2012; Rathore et al., 2013; Stahl et al., 2013). On the other hand, UPR is recruited by the host anti-viral machinery to help eliminating virus infection (Clavarino et al., 2012). Thus UPR is far from being a homeostatic response in terms of virus infection. For viruses that establish persistent infection it is even more important to be able to adapt to chronic ER stress, otherwise diseases will ensue. It is therefore important to understand how HCV interacts with the host UPR, taking into consideration the genotypes and the various experimental systems used to dissect this virus-host interaction.

## Hepatitis C virus

### Life cycle

**Figure 1:**
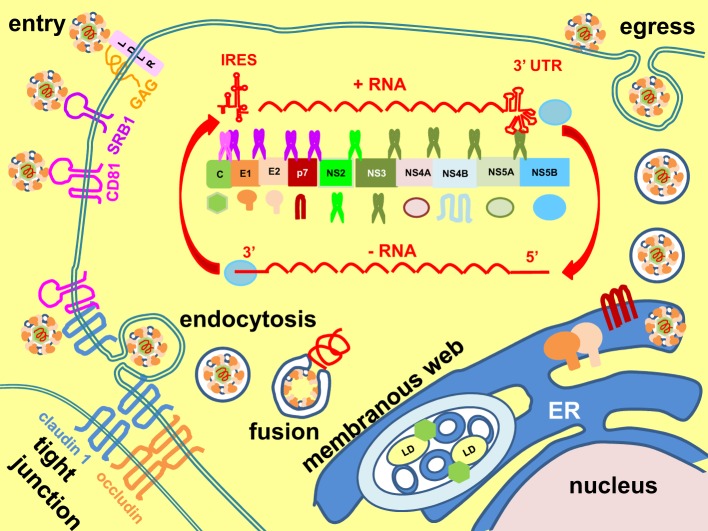
**Hepatitis C virus life cycle**. Hepatitis C virus enters cells by stepwise binding through host receptors low-density lipoprotein receptor (LDLR), glycosaminoglycans (GAGs), scavenger receptor class B member 1 (SRB1), CD81, and the tight junction proteins claudin 1 and occludin. Interaction between CD81 and claudin 1 facilitates viral uptake by clathrin-mediated endocytosis. Endosomal low pH triggers membrane fusion and release of genome into the cytoplasm. The positive-sense (+), single-stranded RNA is translated by an internal ribosome entry site (IRES) element at its 5' untranslated region (UTR) into a single polypeptide, which is then cleaved into the core, E1, E2, p7, NS2, NS3, NS4A, NS4B, NS5A, and NS5B by host signal peptidase (purple scissor) and signal peptide peptidase (pink scissor) and viral autoprotease (NS2-3) (fluorescent green scissor) and serine protease (NS3-NS4A) (dark green scissor). Replication is catalyzed by the RNA-dependent RNA polymerase NS5B, assisted by the helicase activity of NS3, via a negative-sense (−) intermediate RNA (red arrows). Replication takes place in the membranous web, which consists of single, double and multiple membrane vesicles. Formation of the membranous web is induced by NS4B and NS5A. Assembly of virion is initiated on core-coated lipid droplets (LD) followed by budding into the endoplasmic reticulum (ER), where it is coated by the ER-resident envelope proteins E1 and E2. Egress follows the secretory pathway to release the virion to extracellular space. The viroporin, p7, forms ion channels to equilibrate pH gradients during trafficking through the secretory pathway to protect the virion.

HCV is a single-stranded, positive-sense RNA *Hepacivirus* (a genus of the family *Flaviviridae*) with a 9.6 kb genome (Figure 1) (Scheel and Rice, 2013; Simmonds, 2013). The virus particles are unusual in that they are associated with low-density lipoproteins (LDLs) and very low-density lipoproteins (VLDLs) to form the lipoviroparticles (Andre et al., 2002). The virus enters host cells (hepatocytes) by initial binding to low-affinity receptors the LDL receptor and glycosaminoglycans on the heparan sulphate proteoglycans, followed by binding to scavenger receptor class B member 1 and stepwise translocation to post-binding co-receptors the tetraspanin CD81 and tight junction proteins claudin 1 and occludin (Pileri et al., 1998; Agnello et al., 1999; Scarselli et al., 2002; Evans et al., 2007; Ploss et al., 2009; Jiang et al., 2012; Lindenbach and Rice, 2013). Interaction between CD81 and claudin 1 facilitates viral uptake by clathrin-mediated endocytosis (Meertens et al., 2006; Farquhar et al., 2012). Exposure to low pH in the endosome triggers membrane fusion and release of the RNA genome into the cytoplasm (Lavillette et al., 2006). Translation of the RNA genome into a single polypeptide is mediated from an internal ribosome entry site element at the 5' untranslated region (Tsukiyama-Kohara et al., 1992). The polypeptide is then cleaved by the host signal peptidase and signal peptide peptidase and viral autoprotease NS2-3 and serine protease NS3/NS4A co-factor into the structural proteins core, envelopes E1 and E2, and non-structural (NS) proteins p7, NS2, NS3, NS4A, NS4B, NS5A, and NS5B (Hijikata et al., 1991b; Grakoui et al., 1993a; Failla et al., 1994; Lin et al., 1994; Santolini et al., 1994; McLauchlan et al., 2002; Schregel et al., 2009; Scheel and Rice, 2013). Replication takes place in a membranous web, which is a re-organized intracellular membrane structure consisting of single, double, and multiple membrane vesicles (Behrens et al., 1996; Romero-Brey et al., 2012; Bartenschlager et al., 2013; Paul et al., 2013). Formation of the membranous web is mainly induced by NS4B and NS5A (Romero-Brey et al., 2012). Replication is catalyzed by an RNA-dependent RNA polymerase, NS5B, via a negative-sense RNA intermediate, and assisted by the helicase activity of NS3 and host factors cyclophilin A and miR-122 (Behrens et al., 1996; Banerjee and Dasgupta, 2001; Jopling et al., 2005; Kaul et al., 2009). Virion assembly is initiated on core-coated lipid droplets followed by budding into the ER, where the two envelope glycoproteins, E1 and E2, form non-covalently-bonded heterodimers and disulphide-bonded aggregates (Dubuisson et al., 1994; Deleersnyder et al., 1997; Lindenbach and Rice, 2013). Virus particles are released via trafficking through the secretory pathway, where the envelope proteins undergo further glycan modifications and structural re-arrangement into higher ordered oligomeric aggregates (Vieyres et al., 2010, 2014). The viroporin, p7, forms ion channels to equilibrate pH gradients during trafficking through the secretory pathway to protect the virion (Wozniak et al., 2010). Assembly, budding and egress are tightly coupled to host lipoprotein synthesis (Lindenbach and Rice, 2013).

### Genotypes

HCV is classified into 7 genotypes (with >30% sequence variation), sub-divided into sub-types a, b, c, etc. (with 20–25% sequence variation) and then strains/isolates (Simmonds et al., 1994, 2005; Kuiken and Simmonds, 2009). Genotype is a major determining factor in responsiveness to interferon (IFN) treatment and in disease progression in hepatitis C patients (Chayama and Hayes, 2011; Ripoli and Pazienza, 2011). Infection with genotype 1 is more resistant to IFN treatment and presents a more aggressive disease course with the chance of progression into HCC significantly higher. Moreover, HCV in infected patients exists as a population of quasispecies/intrahost variants (Martell et al., 1992; Simmonds et al., 1994, 2005; Holmes, 2010; Domingo et al., 2012). It is anticipated that virus-host interaction is determined at genotypic, sub-genotypic, strain/isolate and even quasispecies/intrahost variants levels. Therefore in this review, we will refer to the genotypes, sub-types and strains/isolates used in various studies.

### HCV experimental systems

**Figure 2:**
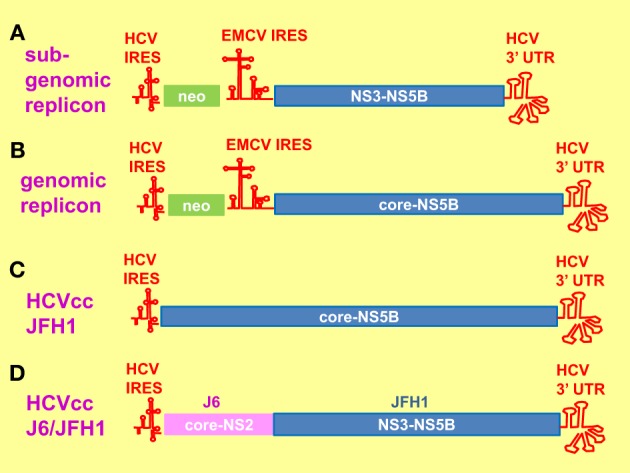
**Hepatitis C virus replication systems. (A)** Sub-genomic replicon (SGR) consists of a bicistronic mRNA. The 5' neomycin (neo) mRNA is translated by the hepatitis C virus (HCV) internal ribosome entry site (IRES) element whereas the 3' mRNA encoding HCV NS3-NS5B plus the 3' untranslated region (UTR) is translated by the encephalomyocarditis virus (EMCV) IRES element. Cell lines harboring the SGR were established by neomycin selection. **(B)** The genomic replicon is similar to that of SGR, apart from that the 3' mRNA encodes core-NS5B plus 3' UTR. **(C)** The HCV cell-cultured infectious system (HCVcc) consists of the entire genomic RNA from JFH1. **(D)** The chimeric J6/JFH1 is created by fusing the core-NS2 from J6 to NS3-NS5B plus 3' UTR from JFH1.

When HCV was discovered in 1989 as the causative agent of post-transfusional non-A, non-B hepatitis, study on the virus was limited to the use of *in vitro* cell-free systems and cell culture expression systems employing transient transfection or viral vectors (Choo et al., 1989; Hijikata et al., 1991b, 1993; Grakoui et al., 1993b). Nevertheless, much has been known about the genomic structure and viral protein functions. *In vivo* study was made possible by the successful infection of chimpanzees by intrahepatic inoculation of the RNA transcript (Kolykhalov et al., 1997). However, the use of chimpanzees is limited and restricted (Mailly et al., 2013). Small animal models have become available by the creation of transgenic mice expressing viral proteins in their livers and chimeric mice with humanized livers (Moriya et al., 1998; Mercer et al., 2001; Dorner et al., 2011). It was not until 1999 when a selectable sub-genomic replicon (SGR) of genotype 1b Con1 isolate was successfully established which allowed the study of the intracellular steps of the virus life cycle (Figure 2A) (Lohmann et al., 1999). Since then some other SGR and genomic replicons have been established (Figure 2B) (Ikeda et al., 2002; Blight et al., 2003; Kato et al., 2003). A pseudotyped virus containing HCV envelope proteins in a retrovirus or lentivirus genomic backbone (HCVpp) was also established to facilitate the study of virus entry (Bartosch et al., 2003). The breakthrough came in 2005 when a cell-cultured infectious system (HCVcc) was established from a wild type genotype 2a JFH1 strain fulminant hepatitis C patient, coupled with derivation of cell lines (Huh7.5, Huh7.5.1) from the parental Huh7 with improved infectivity (Figure 2C) (Lindenbach et al., 2005; Wakita et al., 2005; Zhong et al., 2005). Chimeric viruses were then created by fusing core-NS2 from other genotypes or sub-types to the NS3-5B backbone of JFH1, allowing partial studies of other genotypes (Figure 2D) (Gottwein et al., 2007, 2009; Jensen et al., 2008; Scheel et al., 2008; Li et al., 2011). Currently there has been some success in establishing HCVcc from other genotypes but they all require adaptive mutations, thus do not represent the wild type repertoires (Yi et al., 2006; Date et al., 2012; Li et al., 2012a,b; Ramirez et al., 2014). With the availability of so many systems, therefore in this review, we will refer to the systems and cell lines used in various studies.

## Unfolded protein response

**Figure 3:**
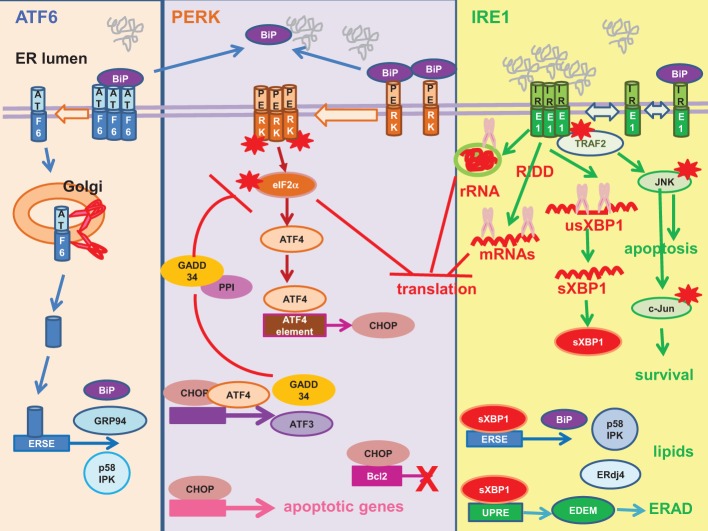
**Unfolded protein response**. Mammalian unfolded protein response (UPR) is a tripartite response involving three proximal sensors: activating factor (ATF) 6, RNA-dependent protein kinase-like ER-resident kinase (PERK) and inositol-requiring enzyme 1 (IRE1). **Left:** ATF6 is sequestered in an inactive state by the molecular chaperone the immunoglobulin heavy-chain binding protein (BiP). Unfolded/malfolded proteins “distract” BiP from ATF6. ATF6 de-oligomerizes and migrates to the Golgi, where the monomer is cleaved by site-1 and site-2 proteases (red scissor) into an active transcription factor. The truncated N-terminal ATF6 is translocated to the nucleus where it transactivates UPR genes harboring an ERSE in their promoters e.g., BiP, glucose-regulated protein 94 (GRP94), P58^IPK^. **Middle:** PERK is sequestered in an inactive state by BiP. Unfolded/malfolded proteins “distract” BiP from PERK, allowing its oligomerization and auto-phosphorylation (red asterisk). The activated PERK then phosphorylates its substrate, the alpha subunit of the eukaryotic initiation factor 2 (eIF2α) (red asterisk) to inhibit global protein synthesis. Paradoxically, translation of ATF4 is upregulated to drive transcription of UPR genes with an ATF4 element in their promoters e.g., the CCAAT/enhancer-binding protein-homologous protein (CHOP). CHOP is a pro-apoptotic transcription factor, as it transactivates a number of apoptotic genes and downregulates the anti-apoptotic Bcl-2. ATF4 co-operates with CHOP to transactivate ATF3 and the growth arrest and DNA damage-inducible protein 34 (GADD34). GADD34 is the regulatory subunit of the protein phosphatase 1 (PP1). It recruits PP1 to dephosphorylate eIF2α (red blunt arrow), thus establishing a negative feedback loop. **Right:** Analogous to yeast, it is thought that IRE1 is activated by direct binding of unfolded/malfolded proteins to its luminal domain and BiP plays a regulatory role. IRE1 possesses endoribonuclease and kinase activity. The endoribonuclease activity mediates unconventional splicing of XBP1 (purple scissor) (usXBP1, unspliced XBP1 mRNA; sXBP1, spliced XBP1 mRNA). The sXBP1 mRNA is translated into an active transcription factor sXBP1 to transactivate genes with ERSE or UPRE in their promoters. XBP1 upregulation of UPR genes such as BiP and ERAD genes such as EDEM and ERdj4 provides a link between UPR and ERAD. XBP1 provides a link between the IRE1 and PERK pathways by upregulating P58^IPK^, an inhibitor of PERK. XBP1 also orchestrates lipogenesis and ER expansion. The other endoribonuclease activity of IRE1 cleaves the ribosomal RNA (rRNA) (purple scissor) and mediates regulated IRE1-dependent decay (RIDD) to cleave a subset of mRNAs (purple scissor) to inhibit protein synthesis. The kinase activity of IRE1 plays a role in cell death/survival. Phosphorylated IRE1 (red asterisk) recruits the adaptor protein tumor necrosis factor receptor-associated factor 2 (TRAF2) to activate a cascade of phosphorylation culminating in pro-apoptotic Jun amino-terminal kinase (JNK) (red asterisk) and pro-survival c-Jun (red asterisk). Red asterisk, activation by phosphorylation.

UPR is a cellular adaptive response for restoring ER homeostasis in response to ER stress (Figure 3) (Walter and Ron, 2011). UPR transduces into a programme of cellular transcriptional and translational responses culminating in upregulation of the molecular chaperone the immunoglobulin heavy-chain binding protein (BiP) to promote protein folding, global inhibition in protein synthesis to reduce protein load and potentiation of ER-associated degradation (ERAD) to eliminate unfolded/malfolded proteins from the ER (Travers et al., 2000; Walter and Ron, 2011).

BiP has been attributed a pivotal role as the master negative regulator of UPR by binding to and repressing the activities of the three proximal UPR sensors: activating transcription factor (ATF) 6, RNA-dependent protein kinase-like ER-resident kinase (PERK), and inositol-requiring enzyme 1 (IRE1) (Bertolotti et al., 2000; Shen et al., 2002a). Accumulation of unfolded/malfolded proteins “ distract” BiP from binding to the UPR sensors. ATF6 de-oligomerizes and migrates to the Golgi where it is cleaved sequentially by site-1 protease and site-2 protease to release an active transcription factor into the nucleus where it transactivates UPR genes harboring an ER-stress element (ERSE) in their promoters e.g., BiP, glucose-regulated protein 94 (GRP94) and P58^IPK^ (Yoshida et al., 1998; Shen et al., 2002a; Nadanaka et al., 2007). P58^IPK^ is an inhibitor of PERK, thus linking the ATF6 pathway to the PERK pathway (Van Huizen et al., 2003).

PERK is an ER stress kinase, activated by dimerisation and autophosphorylation (Harding et al., 1999; Bertolotti et al., 2000). PERK specifically phosphorylates the alpha subunit of the eukaryotic translation initiation factor 2 (eIF2α) causing global inhibition of protein synthesis but paradoxically enhances translation of the transcription factor ATF4 (Harding et al., 2000). ATF4 transactivates UPR genes with an ATF4 element in their promoters e.g., the CCAAT/enhancer-binding protein-homologous protein (CHOP). CHOP is a pro-apoptotic transcription factor owing to its ability to transactivate a number of apoptotic genes and downregulate the anti-apoptotic Bcl-2 (McCullough et al., 2001; Tabas and Ron, 2011). ATF4 and CHOP co-operate to transactivate downstream effectors e.g., ATF3, growth arrest and DNA damage-inducible protein 34 (GADD34) (Han et al., 2013). GADD34 promotes translational recovery by recruiting protein phosphatase 1 to dephosphorylate eIF2α, thus establishing a negative feedback loop (Connor et al., 2001; Novoa et al., 2001, 2003).

IRE1 is a kinase/endoribonuclease activated by self-oligomerisation and autophosphorylation (Sidrauski and Walter, 1997; Bertolotti et al., 2000; Shen et al., 2002a). The IRE1 pathway is an ancient pathway shared with yeast (Tirasophon et al., 1998; Hollien, 2013). In yeast, there is evidence to suggest that IRE1 is activated by direct binding of unfolded protein ligands to its luminal domain whereas BiP plays a regulatory role by maintaining IRE1 oligomeric equilibrium (Credle et al., 2005; Gardner and Walter, 2011; Gardner et al., 2013). The endoribonuclease activity of IRE1 mediates unconventional splicing of the X-box binding protein 1 (XBP1) for its productive translation into an active, multi-functional transcription factor, the spliced XBP1 (sXBP1) (Calfon et al., 2002). sXBP1 transactivates ERSE in the promoters of UPR genes and the mammalian UPR element (UPRE) in the promoters of ERAD genes, thus providing a link between UPR and ERAD (Yoshida et al., 2003). Indeed, UPRE-mediated transcriptional induction of the ER degradation-enhancing α-mannosidase-like protein (EDEM) is directly involved in the recognition of malfolded proteins for degradation. Another protein ERdj4 transactivated by sXBP1 also participates in ERAD (Shen et al., 2002b; Lee et al., 2003; Lai et al., 2012). Similar to that of ATF6, XBP1 also links the IRE1 pathway to the PERK pathway by upregulating the inhibitor of PERK, P58^IPK^, to aid in translational recovery (Yan et al., 2002; Lee et al., 2003; Van Huizen et al., 2003). XBP1 also assumes additional function in the regulation of lipogenesis and ER expansion (Lee et al., 2008; Glimcher and Lee, 2009; Brewer and Jackowski, 2012). The endoribonuclease activity of IRE1 also participates in translational repression by cleavage of the 28S ribosomal RNA and a subset of mRNAs via regulated IRE1-dependent decay (Iwawaki et al., 2001; Hollien et al., 2009). On the other hand, the kinase activity of IRE1 regulates cell death/survival (Urano et al., 2000; Tabas and Ron, 2011). Phosphorylated IRE1 associates with the adaptor protein tumor necrosis factor receptor-associated factor 2 to initiate a cascade of phosphorylation culminating in that of the pro-apoptotic Jun amino-terminal kinase (JNK) and pro-survival c-Jun (Darling and Cook, 2014).

## Evidence of UPR in hepatitis C

There is as yet no consistent clinical data to support or refute the presence of ER stress in hepatitis C patients (Asselah et al., 2010; McPherson et al., 2011). Comparison between HCV-positive and -negative liver biopsy using real-time RT-PCR did not reveal any significant variation in the mRNA levels of GRP94, sXBP1 and EDEM (McPherson et al., 2011). Immunohistochemistry also did not detect any overall difference in the intensity of BiP between chronic hepatitis C and non-diseased livers, however, the staining was variable and one HCV sample showed a very high level of BiP. This may be explained by HCV being a focal infection, infecting only 7–20% of the liver (Liang et al., 2009; Stiffler et al., 2009). As a result, random sampling may not be able to detect a significant change in the mRNA/protein level in an area of mixed infected- and uninfected-hepatocytes. Indeed, using electron microscopy, dilated and disorganized ER indicative of ER stress was observed in hepatocytes from liver biopsy of mild chronic hepatitis C patients (Asselah et al., 2010). Evidence of ER stress in these liver samples was further confirmed using Western blotting which showed marked elevation in the levels of the proximal sensors ATF6α, ATF6β, sXBP1, and phosphorylated PERK and select subsets of downstream effectors BiP, phospho-eIF2α, ATF4, and EDEM. A study on a cohort of HCV HCC patients also demonstrated increased UPR markers of sXBP1, BiP, and ATF6 in liver biopsy by using immunohistochemistry and Western blotting (Shuda et al., 2003). It is therefore essential that concrete clinical evidence should await the use of more sensitive methods to detect, at single cells level, co-localization of ER stress markers in infected cells as compared to neighboring uninfected cells. Nevertheless, there is overwhelming evidence from *in vivo* and *in vitro* experiments to suggest that the ER stress response plays an important role in the life cycle of HCV (Liberman et al., 1999; Tardif et al., 2002, 2004; Benali-Furet et al., 2005; Chan and Egan, 2005, 2009; Ciccaglione et al., 2005, 2007; Zheng et al., 2005; Tumurbaatar et al., 2007; Sekine-Osajima et al., 2008; Joyce et al., 2009; Li et al., 2009; Mishima et al., 2010; Von Dem Bussche et al., 2010; Funaoka et al., 2011; Merquiol et al., 2011; Shinohara et al., 2013). Importantly, by using immunohistochemistry and confocal microscopy, increased level of the UPR marker, BiP, was found to co-localize with HCV-infected hepatocytes in SCID/Alb/uPA mice (chimeric mice with humanized livers) infected with genotype 1a H77 or intrahepatically inoculated with H77 RNA (Joyce et al., 2009). Infection of humanized mice with another genotype (2a) JFH1 strain also resulted in increased levels of BiP and CHOP in the livers (Mishima et al., 2010). Further *in vivo* evidence of ER stress was obtained in transgenic mice stably expressing the entire open reading frame, the core protein or inducibly expressing C-E1-E2-p7 in the livers (Benali-Furet et al., 2005; Tumurbaatar et al., 2007; Merquiol et al., 2011). Modulation of the UPR was widely observed in tissue-cultured hepatocytes infected with HCV; in cells harboring the HCV genomic replicon and SGR and in cells ectopically expressing individual viral proteins (Liberman et al., 1999; Tardif et al., 2002, 2004; Benali-Furet et al., 2005; Chan and Egan, 2005, 2009; Ciccaglione et al., 2005, 2007; Zheng et al., 2005; Sekine-Osajima et al., 2008; Li et al., 2009; Von Dem Bussche et al., 2010; Funaoka et al., 2011; Shinohara et al., 2013).

## UPR signaling in hepatitis C

Some viruses can selectively activate or suppress one or more of the UPR tripartite pathways to facilitate their own replication (Isler et al., 2005; Smith et al., 2006; Yu et al., 2006; Jheng et al., 2010; Ambrose and Mackenzie, 2011; Pena and Harris, 2011; Burnett et al., 2012; Galindo et al., 2012; Qian et al., 2012; Rathore et al., 2013; Stahl et al., 2013). It is apparent that HCV infection activates all three proximal sensors (Ke and Chen, 2011; Merquiol et al., 2011). Infection of the hepatocyte sub-line Huh7.5.1 with JFH1 (2a) induced an acute ER stress peaking at 2–5 days post-infection (dpi), concomitant with phosphorylation of IRE1, eIF2α, and JNK, XBP1 splicing, ATF6 cleavage and upregulation of GADD34, ERdj4, P58^IPK^, ATF3, ATF4, and CHOP (Merquiol et al., 2011). It then subsided into a chronic and milder ER stress response persisting up to 14 dpi, with elevated mRNA levels of CHOP, ATF3, sXBP1, and P58^IPK^ and increased level of phospho-eIF2α. ER stress response is not restricted to the sub-line Huh7.5.1, as infection of the parental lines Huh7 or Huh7.5 with JFH1 (2a) also induced ER stress (Ke and Chen, 2011). Infection of Huh7 with JFH1 provoked an acute ER stress response concomitant with ATF6 cleavage, XBP1 splicing and PERK phosphorylation at 6–9 dpi followed by a chronic and milder ER stress with a diminished CHOP level at 15–22 dpi. Similarly, infection of Huh7.5 with JFH1 (2a) has been shown to transactivate the Bip, CHOP, and ATF6 promoters (Von Dem Bussche et al., 2010).

Currently *in vitro* infection study with wild type genotype is only achievable with the strain JFH1 and yet JFH1 was isolated from a patient with fulminant hepatitis-a rare manifestation of HCV diseases (Wakita et al., 2005; Lohmann and Bartenschlager, 2014). It is therefore important that studies should be extended to other genotypes before it can be generalized that ER stress is a common phenomenon of chronic hepatitis C. Chimeric HCV has been created by fusing the structural proteins from all seven genotypes with the NS proteins of JFH1, which should at least allow us to study the role of genotypic structural proteins in UPR (Gottwein et al., 2007, 2009; Jensen et al., 2008; Scheel et al., 2008; Li et al., 2011). An intragenotypic chimera J6/JFH1 has already been shown to be capable of eliciting the UPR, as evident by the increased levels of CHOP and sXBP1 at 1–3 dpi (Mohl et al., 2012). Some success has been achieved to establish cell-cultured infectious systems for genotypes 1a (H77 and TN), 1b (NC1), 2a (J6), and 2b (J8, DH8, DH10) but they require a number of adaptive mutations (Yi et al., 2006; Date et al., 2012; Li et al., 2012a,b; Ramirez et al., 2014). At the moment, studies with wild type genotypes other than JFH1 still rely on the use of genomic replicons (Benali-Furet et al., 2005; Shinohara et al., 2013). Similar to that in JFH1-infected Huh7, all three pathways have been activated in Huh7 cells harboring a genomic replicon of genotype 1b O strain, as indicated by the phosphorylation of eIF2α, XBP1 splicing and increased levels of IRE1, phospho-JNK, and phospho-c-Jun (Shinohara et al., 2013).

## Which viral proteins mediate UPR?

A number of steps in the virus life cycle are potential trigger of ER stress e.g., the maturation of the viral envelope glycoproteins in the ER, formation of replication complex on the ER, virus assembly, and budding of virus particles into the ER (Scheel and Rice, 2013).

### Envelope proteins

SGR (devoid of C-E1-E2-p7-NS2) is generally less capable (or incapable) of triggering the UPR than its full-length counterpart, suggesting that the main contributors to ER stress lie within the structural-NS2 region (Von Dem Bussche et al., 2010; Mohl et al., 2012). A genomic replicon devoid of the envelope proteins E1 and E2 failed to elicit UPR in transfected Huh7 cells, implicating a pivotal role of the envelope proteins in the elicitation of ER stress (Mohl et al., 2012). This is consistent with the ER residence of the envelope proteins. Using transient transfection of envelope proteins to physiological levels, we have confirmed that the HCV envelope proteins are capable of inducing the UPR in hepatocytes HepG2 and Huh7 as well as non-hepatocyte HeLa (Chan and Egan, 2005, 2009).

### Core

The core protein, which does not enter the ER lumen but is important in lipid droplet formation and virus assembly and budding, also elicits the UPR (McLauchlan et al., 2002; Benali-Furet et al., 2005; Funaoka et al., 2011; Scheel and Rice, 2013). Evidence of ER stress has been documented in tissue-cultured cells transfected with the HCV-core and in the livers of HCV-core transgenic mice (Benali-Furet et al., 2005). The significant role of the core protein can be illustrated by the considerable effects of mutating the core residues R70Q, R70H, L91M on the UPR in Huh7 cells infected or transfected with JFH1 (Funaoka et al., 2011).

### NS2

NS2 does not enter the ER lumen despite being a transmembrane protein (Bartenschlager et al., 2013). Apart from harboring a protease, NS2 is important in organizing the virus assembly complex (Lindenbach and Rice, 2013). One study implicated a major role of NS2 in provoking the UPR based on the detection of increased BiP in Huh-7 cells transfected with core-E1-E2-p7-NS2 compared with that transfected with core-E1-E2-p7, however, expression of NS2 was barely detectable (Von Dem Bussche et al., 2010). Whereas the increases of BiP at the promoter and mRNA levels were very modest, it is not clear why the more pronounced increase of BiP protein level necessitated detection by immunoprecipitation-Western blotting rather than the more straightforward Western blotting. Ectopic expression of NS2 from genotype 1a in Huh7 cells resulted in eIF2α phosphorylation and modest increases of BiP, CHOP, and ATF6 at the promoter and mRNA levels together with a more pronounced increase in BiP protein level (again, detected by immunoprecipitation-Western blotting). In contrast, another hepatocyte cell line Hep3B stably expressing NS2 from genotype 1b (k isolate) failed to induce ATF6 cleavage (Li et al., 2009). Therefore, whether NS2 is the main contributor of ER stress, as claimed, still needs robust testing (Von Dem Bussche et al., 2010).

### NS4B

Huh7 cells harboring SGR were capable of inducing ATF6 cleavage and XBP1 splicing but suppressing the downstream activation of UPRE and EDEM by sXBP1 (Tardif et al., 2002, 2004). This suppressive effect could be attributed to NS4B as ectopic expression of NS4B in Huh7 cells displayed a similar pattern of ATF6 cleavage and XBP1 splicing without downstream activation of EDEM (Zheng et al., 2005; Li et al., 2009). NS4B is important in membranous web/replication complex formation (Bartenschlager et al., 2013). Similar to NS2, it also does not enter the ER lumen despite being a transmembrane protein. The role of NS4B in UPR could be modulating. Indeed, ERAD activation, as demonstrated by XBP1 splicing and upregulation of EDEMs mRNAs, clearly exists during infection of Huh7.5.1 with JFH1 (Saeed et al., 2011). EDEM interaction with E1 and E2 resulted in ubiquitination of E2 and decrease in virus particle production. This is not ideal for the virus. Subsequently, NS4B may act to modulate the UPR by suppressing the ERAD to help damping down the inhibitory effect of EDEM in order to regulate and fine-tune virus particle production.

### NS5A/5B

NS5A/5B are integral to viral replication (Scheel and Rice, 2013). Infection of humanized mice with NS5A/5B mutants of JFH1 led to increased expression of BiP and CHOP, suggesting a role of the NS5 proteins in ER stress although it is not clear whether they act directly or indirectly (Mishima et al., 2010). It is possible that the enhancing effect of the NS5 mutants on the UPR may be an indirect result of a higher replication rate of these mutants leading to increased production of the responsible proteins i.e., core, E1, E2. Whether the NS5 proteins directly induce the UPR still needs to be shown but Hep3B cells stably expressing NS5B genotype 1b (k isolate) failed to induce ATF6 cleavage (Li et al., 2009).

## How do envelope proteins elicit UPR?

**Figure 4:**
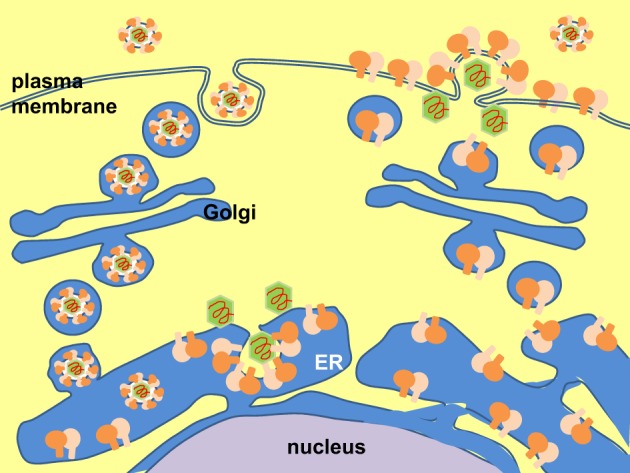
**Two modes of virus budding. Left:** Virus budding into the endoplasmic reticulum (ER). Virion assembles and buds into the ER, where it is coated by the ER-resident envelope proteins. Egress of virus particle follows the host secretory pathway and released into the extracellular space. **Right:** Virus budding from the plasma membrane. Envelope proteins are targeted to the ER and transported to the cell surface via the host secretory pathway. Virion assembles and buds through the plasma membrane.

Enveloped viruses either bud through the plasma membrane or an intracellular compartment e.g., ER (Figure 4) (Garoff et al., 2004; Stertz et al., 2007; Murakami, 2012; Prange, 2012; Vieyres et al., 2014). In either case, the envelope proteins will be first targeted to the ER for post-translational modification and maturation. Many viral envelope proteins are significant inducers of UPR, whether they are ER-resident proteins (for viruses budding into the ER) or are just trafficking through the ER *en route* to the plasma membrane (for viruses budding through the plasma membrane) (Dimcheff et al., 2003, 2004; Wang et al., 2003, 2006; Hsieh et al., 2004; Liu et al., 2004, 2006; Nanua and Yoshimura, 2004; Qiang et al., 2004; Chua et al., 2005; Chan et al., 2006; Yoshimura and Luo, 2007; Yoshimura et al., 2008; Zhao and Yoshimura, 2008; Favreau et al., 2009; Portis et al., 2009; Barry et al., 2010; Dediego et al., 2011; Hung et al., 2011).

### Mutant envelope proteins as UPR inducers

Several mutated viral envelope proteins are significant UPR inducers and major determinants of virulence, in analogous to many human diseases which are caused by retention of mutated cellular proteins in the ER e.g., the genetic variant null Hong Kong of α1-antitrypsin and the ΔF508 cystic fibrosis transmembrane conductance regulator (Oda et al., 2003; Gnann et al., 2004). Retrovirus buds through the plasma membrane (Murakami, 2012). Virulent strains of retrovirus harbor mutations in the envelope proteins resulting in retention of inefficiently folded envelope proteins in the ER, leading to elicitation of the UPR which is a major determinant of neurovirulence (Dimcheff et al., 2003, 2004; Liu et al., 2004, 2006; Nanua and Yoshimura, 2004; Qiang et al., 2004; Yoshimura and Luo, 2007; Yoshimura et al., 2008; Zhao and Yoshimura, 2008; Portis et al., 2009). Hepatitis B virus buds into the ER-Golgi intermediate or other intracellular compartments, mutations in the large surface protein resulted in ER retention, provoking ER stress which is associated with hepatocarcinogenesis (Wang et al., 2003, 2006; Hsieh et al., 2004; Chua et al., 2005; Hung et al., 2011; Prange, 2012). Coronavirus also buds into the ER-Golgi intermediate compartment (Garoff et al., 1998; Stertz et al., 2007). Its spike protein is an UPR inducer (Chan et al., 2006; Versteeg et al., 2007; Siu et al., 2014). Mutations in the spike protein have been associated with persistence and translational attenuation and these mutations have also been found to enhance UPR, cytotoxicity and cell death and confer neurovirulence (Favreau et al., 2009).

### Immature virion as UPR inducer

For viruses that bud into the ER it is not clear how the ER-residing envelope proteins will induce UPR. Many of these envelope proteins will undergo further processing and re-organization/conformational changes after incorporation into the immature virion and trafficking through the secretory pathway. In flavivirus, 60 trimeric prM/E assemble as immature virion in the ER (Pierson and Diamond, 2012). During transit through the Golgi E undergoes dramatic re-organization and collapses onto the virion surface whereas a cleavage site on prM is exposed for furin proteolysis. Flaviviruses are prolific inducers of UPR (Jordan et al., 2002; Su et al., 2002; Yu et al., 2006; Medigeshi et al., 2007; Umareddy et al., 2007; Ambrose and Mackenzie, 2011; Klomporn et al., 2011; Paradkar et al., 2011; Pena and Harris, 2011; Wu et al., 2011; Ambrose and Mackenzie, 2013; Blazquez et al., 2013; Yu et al., 2013; Bhattacharyya et al., 2014). UPR has been documented in infections of Dengue virus, West Nile virus, Japanese encephalitis virus, tick-borne encephalitis virus and Usutu virus. The HCV envelope proteins are synthesized as part of a single polypeptide (Grakoui et al., 1993b). After importing into the ER by signal peptides at their respective N-termini, they are cleaved into E1 and E2 by cellular signal peptidase (Hijikata et al., 1991a; Lin et al., 1994). Inside the ER, E1 and E2 form two types of complexes: non-covalently-bonded E1-E2 heterodimer and disulphide-bonded aggregates, neither is the mature form (Dubuisson et al., 1994; Dubuisson and Rice, 1996; Deleersnyder et al., 1997). It is not clear which of these forms of E1E2 is acquired by the virion when the virus buds into the ER as E1E2 undergo further conformational changes into aggregated oligomers when the virus particles transit through the secretory pathway (Vieyres et al., 2010, 2014). There has been evidence to suggest that HCV envelope proteins are major UPR inducers (Mohl et al., 2012). Alphavirus does not bud through the ER and yet its maturation resembles that of flavivirus in that the immature prE2/E1 trimer assembled in the ER undergoes furin cleavage in the Golgi into E3(=pr)/E2/E1 trimer (Garoff et al., 2004; Vaney et al., 2013). UPR has been documented in infections of Chikungunya virus and Sindbis virus (Joubert et al., 2012; Abraham et al., 2013; Rathore et al., 2013). The envelope proteins of Semliki Forest virus have been shown to be responsible for the induction of UPR (Barry et al., 2010). Therefore, we speculate that the immature ER form may be in itself a trigger of the UPR, irrespective of whether the virions bud through the ER or other sites.

### How do HCV envelope proteins activate UPR?

**Figure 5:**
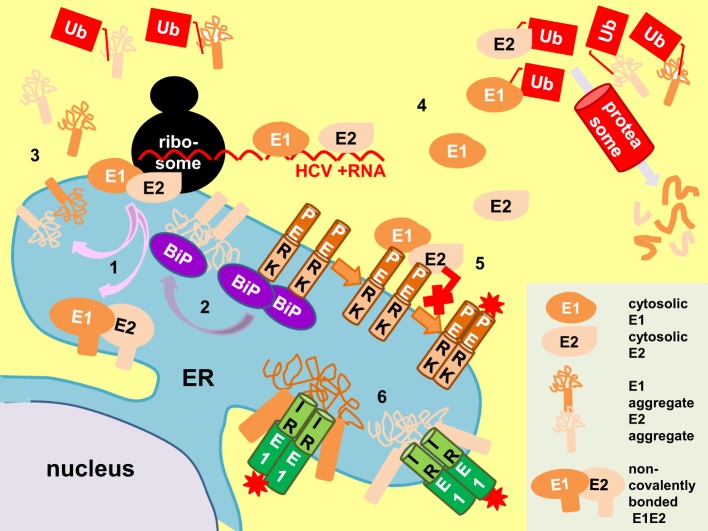
**Proposed mechanisms of E1/E2 activation of UPR**. E1 and E2 are targeted and mature in the endoplasmic reticulum (ER) to form non-covalently-bonded heterodimers and disulphide-bonded aggregates (pink arrows, 1). The E2 aggregates distract BiP from PERK (purple arrow), allowing PERK oligomerization and activation (brown arrows, 2). Retrotranslocated (3) and cytosolic E1/E2 from surplus protein synthesis (4) can also activate the unfolded protein response (UPR) by perturbation of proteasomal function (Ub, polyubiquitin). Conversely, cytosolic E1/E2 can bind to the cytoplasmic domain of PERK and inhibit its activation (red cross, 5). Direct binding of E1 and E2 aggregates to the luminal domain of IRE1 can also activate UPR (6). Red asterisk, activation by phosphorylation.

E1 and E2 accumulate in the ER, placing them in proximity to interact with BiP (Choukhi et al., 1998). Folding of E1 and E2 into the non-covalently-bonded heterodimer utilizes the canonical chaperone calnexin and calreticulin whereas the E2 aggregates are bound by BiP (Figure 5) (Dubuisson and Rice, 1996; Choukhi et al., 1998). This may explain why E2 was able to elicit the UPR (Liberman et al., 1999). However, Bip binds to the E1 aggregates inefficiently or not at all, leading to the possibility that E1 may induce UPR by other means (Choukhi et al., 1998; Liberman et al., 1999; Merola et al., 2001).

One plausible mechanism is that E1 (or even E2) induces UPR by impairing ERAD as ERAD and UPR exist in a regulatory loop (Travers et al., 2000). It is well known that cytosolic proteins such as the polyQ aggregates induce UPR by perturbation of proteasomal degradative function (Friedlander et al., 2000; Travers et al., 2000; Nishitoh et al., 2002). We have shown ERAD engagement in cells transfected with E1 and/or E2 by the demonstration of XBP1 splicing and UPRE induction in these cells (Chan and Egan, 2005). Although E1/E2 mature in the ER, it is possible that some of them have been directed to the cytoplasm during synthesis or as a result of retro-translocation from the ER. There is *in vivo* evidence of retrograde transport of E1 from the ER to the cytoplasm for proteasome degradation based on the detection of a deglycosylated-deamidated T-epitope from an HCV-infected chimpanzee (Selby et al., 1999). Cytosolic existence of E2 has been demonstrated in vector-expression system although it still yet has to show the cytosolic existence of E2 in infected cells (Pavio et al., 2002). By removing the signal peptides from E1 and E2 we re-directed expression of these proteins to the cytoplasm (Egan et al., 2013). These cytosolic-targeting E1/E2 did not induce UPR. Instead, they repressed tunicamycin-induced UPR possibly as a result of binding to the cytoplasmic domain of PERK and blocking its activation, suggesting that UPR induction by ERAD perturbation is unlikely (Pavio et al., 2003; Egan et al., 2013).

Another possibility is that E1 (or even E2) can trigger UPR by direct binding to one or more of the UPR sensors in the ER lumen. This is especially true when UPR triggered by many virus infections is often skewed suggesting a canonical tripartite-responsive BiP derepression mechanism may not be sufficient to explain these skewed UPR in cases of virus infections. Studies with yeast have shown that UPR can be triggered by direct binding of unfolded proteins to the luminal domain of IRE1 (Credle et al., 2005; Gardner and Walter, 2011; Gardner et al., 2013). The luminal domain of PERK bears secondary structure homology with that of IRE1, by extrapolation, direct binding of unfolded proteins to PERK can also be feasible (Gardner et al., 2013). Direct binding between the herpes simplex virus glycoprotein B and the luminal domain of PERK has been documented but in this case, binding results in repression rather than elicitation of the UPR (Mulvey et al., 2007). Toxic lipids are directly sensed by the transmembrane domains of IRE1 and PERK to provoke the UPR, further supporting the idea that mechanisms other than BiP derepression is possible (Volmer et al., 2013).

## How do core and NS proteins elicit UPR?

During polyprotein processing, the signal peptide at the C terminus of the core protein directs the translocation of E1 into the ER, after that the signal peptidase will cleave at the C-terminal end of the core protein at amino acid (aa) residue 191 (Santolini et al., 1994). This intermediate core protein is anchored onto the cytosolic side of the ER membrane by a membrane anchor. Maturation of the core protein involves another intramembrane cleavage event at aa173–182 by signal peptide peptidase in the ER membrane (Okamoto et al., 2008a; Pene et al., 2009). The exact C terminus has not been determined but a minimum of 177 aa residues seems to be required for productive virus production (Kopp et al., 2010). The mature core protein is then released from the ER to traffic to lipid droplets to orchestrate virus assembly (McLauchlan et al., 2002). It appears that the core protein is never directed inside the ER to be able to interact with BiP to trigger the canonical UPR signaling. This is also true for the NS proteins which do not appear to enter the ER lumen even though NS2 and NS4B are transmembrane proteins (Romero-Brey et al., 2012). The question remains how then can their cytosolic presence elicit the UPR?

### Proteasomal perturbation

**Figure 6:**
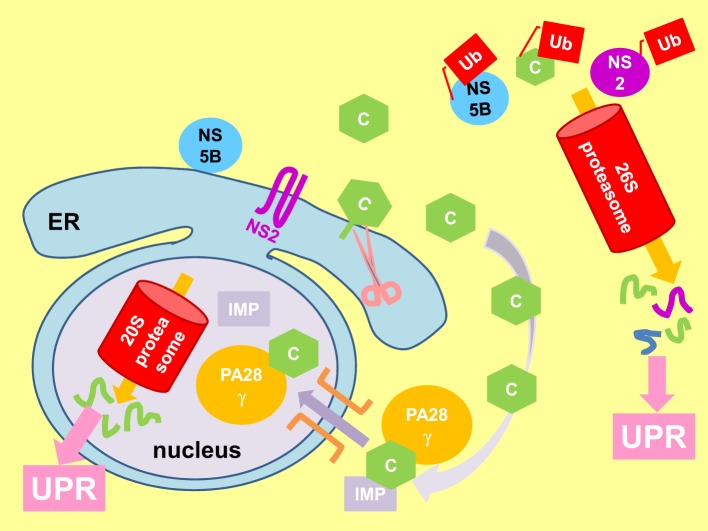
**Viral proteins perturb proteasomal function to elicit UPR**. Immature core protein (C) attaches to the cytosolic side of the endoplasmic reticulum (ER) membrane by a membrane anchor. Cleavage of the membrane anchor (pink scissor) releases mature core to the cytoplasm where it can be polyubiquitinated (Ub) and degraded by the cytosolic 26S proteasome. The mature core protein can also be imported into the nucleus by association with the proteasome activator PA28γ and importin (IMP) (purple arrow), where it is degraded by the 20S proteasome independent of ubiquitin. Also shown is the polyubiquitination and degradation of non-structural protein 2 (NS2) and NS5B by the 26S proteasome. Perturbation of proteasomal function elicits the unfolded protein response (UPR).

Perturbation of proteasomal activity is one possibility (Friedlander et al., 2000; Travers et al., 2000; Nishitoh et al., 2002). The core, NS2 and NS5B proteins have been shown to interact with the proteasomal pathways (Figure 6) (Gao et al., 2003; Moriishi et al., 2003; Franck et al., 2005; Shirakura et al., 2007; Suzuki et al., 2009). Moreover, interaction of the core protein with the proteasome activator PA28γ is responsible for the pathogenesis of steatosis, HCC and other liver pathology in core-transgenic mice and virus propagation in JFH1-infected Huh7 cells (Moriishi et al., 2007, 2010; Tripathi et al., 2012).

### Perturbation of membranous web protein chaperone activity

**Figure 7:**
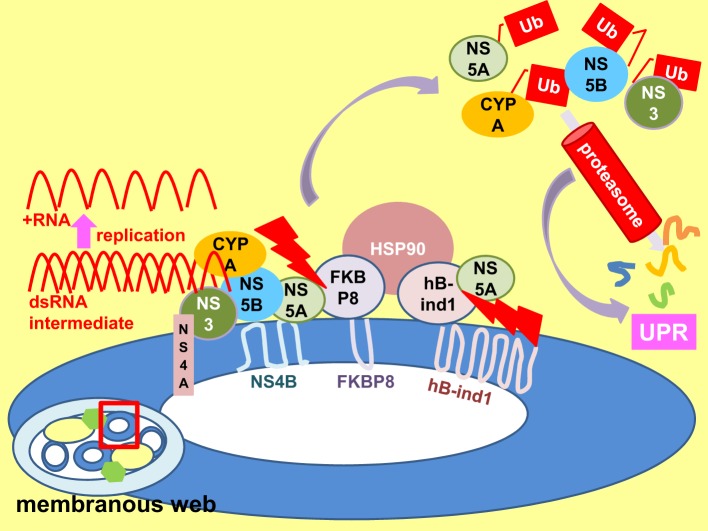
**Perturbation of replicase chaperone activity results in UPR**. An enlarged view of a membrane vesicle (enclosed by a red square) within the membranous web. Heat shock protein 90 (HSP90) chaperones folding of viral non-structural (NS) 3-NS5B proteins and host protein cyclophilin A (CYPA) etc. in the replication complex to facilitate viral replication. HSP90 forms a complex with NS5A via interactions with its co-chaperone FK506-binding protein 8 (FKBP8) and the human butyrate-induced transcript 1 (hB-ind1). Disruption of co-chaperone-NS5A interaction (red serrated arrows) interrupts HSP90 chaperone activity, resulting in increased degradation of polyubiquitinated (Ub) proteins and perturbation of proteasomal function, leading to the unfolded protein response (UPR) (purple arrows).

The cytosolic chaperone heat shock protein 90 (HSP90) promotes HCV replication by facilitating host and viral protein folding in the replication complex of the membranous web (Figure 7) (Taguwa et al., 2009). Inhibition of HSP90 activity reduces protein folding, accelerates proteasome degradation and induces the UPR. HSP90 is recruited into the replication complex by means of interaction between its co-chaperones FK506-binding protein 8 (FKBP8) and the human butyrate-induced transcript 1 (hB-ind1) and NS5A (Okamoto et al., 2006; Taguwa et al., 2008). It is therefore possible that any changes in HSP90-FKBP8/hB-ind1-NS5A interaction can disrupt the chaperone activity of HSP90 leading to UPR. Indeed, interaction of NS5A with FKBP8 has already been implicated in pathogenesis via activation of mammalian target of rapamycin anti-apoptotic function (Peng et al., 2010). In contrast, a JFH1 NS5A/5B multiple mutants exhibited a higher replication rate and yet provoking a stronger UPR in humanized mice (Mishima et al., 2010). However, the sample size is small (only one mouse from each of the test and control groups was dissected for the UPR markers). Moreover, the mutations are not likely to be involved in co-chaperone binding, suggesting another mechanism of UPR regulation. Previously, it has been found that mutation of a single amino acid V/I121A in NS5A is sufficient to abolish its interaction with FKBP8 impairing virus replication (Okamoto et al., 2008b). It would be interesting to see whether this mutation will provoke a stronger UPR.

### Direct binding to cytosolic domains of UPR sensors

**Figure 8:**
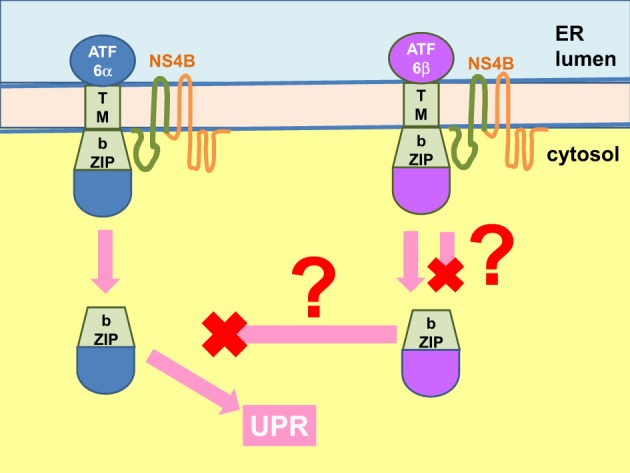
**NS4B interacts with ATF6 to modulate UPR**. The hepatitis C virus (HCV) non-structural (NS) 4B protein binds to the b-ZIP and transmembrane (TM) domains of the activating factor 6 (ATF6) α and β (binding domains shown in green for both NS4B and ATF6). Binding of ATF6α likely triggers its Golgi translocation and cleavage into an active transcription factor to mediate the unfolded protein response (UPR). The fate of NS4B binding to ATF6β is unknown. It can either trigger or inhibit ATF6β Golgi translocation and activation. The end result could be modulation of the UPR but whether ATF6β is an inhibitor of ATF6α is controversial. ER, endoplasmic reticulum.

Another possibility for cytosolic proteins to trigger UPR is by direct binding to the cytoplasmic domain of the UPR sensors. Currently there is no evidence for interaction of the core protein with any of the cytosolic domains of the UPR sensors. NS4B, however, interacts with the bZIP motif of ATF6β via a predicted bZIP motif in its N-terminal cytoplasmic domain although there is as yet no functional analysis of whether this interaction leads to induction or suppression of ATF6β activity (Figure 8) (Tong et al., 2002; Welsch et al., 2007). On the other hand, NS4B also interacts with ATF6α, despite to a lesser extent, and it is plausible that this interaction signals ATF6α cleavage as observed in hepatocytes and non-hepatocytes expressing NS4B (Tardif et al., 2002; Tong et al., 2002; Zheng et al., 2005; Li et al., 2009). However, expression of NS4B alone also induced XBP1 splicing, suggesting that at least one other mechanism is operating to induce the UPR (Li et al., 2009).

### Lipid perturbation

**Figure 9:**
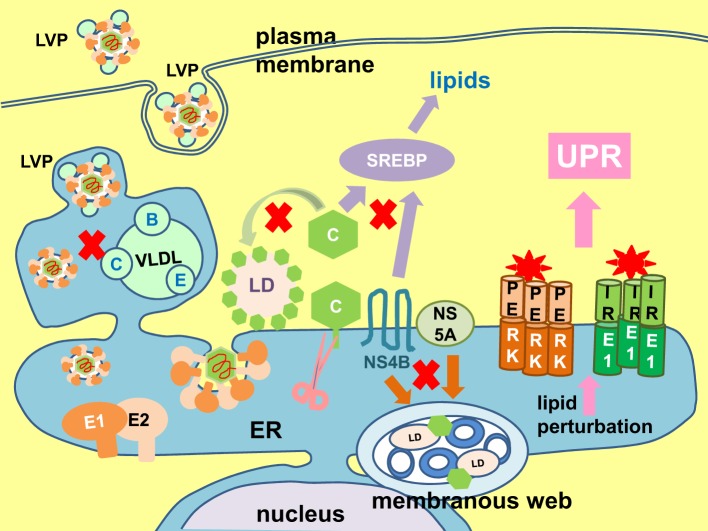
**Lipid perturbation elicits unfolded protein response**. The hepatitis C virus (HCV) core protein (C) plays a part in lipid droplet (LD) formation (green arrow), and also coats the lipid droplet to initiate virion assembly and budding into the envelope proteins E1- and E2-coated endoplasmic reticulum (ER). Maturation and egress of the virion is tightly coupled to host very low density lipoprotein (VLDL) synthesis (A, apolipoprotein A; C, apolipoprotein C; E, apolipoprotein E), resulting in the formation of a lipoviroparticle (LVP). The non-structural (NS) 4B and NS5A proteins induce formation of the membranous web (brown arrow). The core and NS4B proteins have been shown to transactivate the sterol regulatory element binding protein (SREBP), the master regulator of lipogenesis (purple arrow). Perturbation in any of these lipid synthesis pathways will easily signal to elicit the unfolded protein response (UPR) (pink arrow). Putative lipid metabolic steps vulnerable to perturbation are marked with red crosses.

UPR regulates lipogenesis and ER membrane expansion (Lee et al., 2008; Glimcher and Lee, 2009; Brewer and Jackowski, 2012). On the contrary, lipid perturbation is one of the triggers of UPR (Volmer et al., 2013). HCV virus particle formation is intimately coupled to the host lipogenesis (Figure 9). The core protein targets to the lipid droplets which is the initial site of virus assembly (McLauchlan et al., 2002; Lindenbach and Rice, 2013). Virus infection also induces massive intracellular membrane re-organization to form the membranous web as the site of virus replication (Behrens et al., 1996; Romero-Brey et al., 2012; Bartenschlager et al., 2013; Paul et al., 2013). Budding of virion into the ER and egress through the secretory is tightly linked to lipoprotein synthesis (Lindenbach and Rice, 2013). Finally, the virus particle associates with lipoproteins to form lipoviroparticle which is essential for virus infectivity (Andre et al., 2002; Felmlee et al., 2013). Therefore, throughout the life cycle of the virus, there is a constant need for lipids and lipoproteins. Transactivation of the sterol regulatory element binding proteins, the master regulator of lipogenesis, has been observed in Huh7 cells infected with JFH1 (2a) or harboring a SGR and in cells ectopically expressing the core protein or NS4B (Waris et al., 2007; Rahman et al., 2009). It is not difficult to imagine that this can easily lead to perturbation of lipid homeostasis and trigger the UPR. Indeed, hepatitis C patients exhibit many lipid and lipoprotein metabolism disorders such as hepatic steatosis (fatty liver), hypobetalipoproteinaemia, and hypocholesterolemia (Serfaty et al., 2001; Colloredo et al., 2004; Felmlee et al., 2013). Experimentally, both exogenous and endogenous sources of fatty acids were capable of inducing ER stress in Huh7 cells infected with JFH1 or harboring a SGR (Rahman et al., 2009; Gunduz et al., 2012).

## Concluding remarks

Despite overwhelming evidence from *in vivo* (transgenic and humanized mice) and *in vitro* studies to indicate that HCV infection causes ER stress and induces the UPR, we still need to confirm the presence of ER stress in hepatitis C patients by conducting clinical studies at single cells level (Liberman et al., 1999; Tardif et al., 2002, 2004; Benali-Furet et al., 2005; Chan and Egan, 2005, 2009; Ciccaglione et al., 2005, 2007; Zheng et al., 2005; Tumurbaatar et al., 2007; Sekine-Osajima et al., 2008; Joyce et al., 2009; Li et al., 2009; Mishima et al., 2010; Von Dem Bussche et al., 2010; Funaoka et al., 2011; Merquiol et al., 2011; Shinohara et al., 2013). Clinical data will need to be further corroborated and elaborated using well-controlled experiments. Genotype is a major determinant of IFN responsiveness and disease progression, therefore, it is important that studies should be extended to other genotypes before it can be generalized that ER stress is a common phenomenon of chronic hepatitis C (Chayama and Hayes, 2011; Ripoli and Pazienza, 2011).

A number of steps in the virus life cycle are potential trigger of ER stress e.g., the maturation of the viral envelope glycoproteins in the ER, formation of replication complex on the ER and virus assembly and budding of virus particles into the ER (Scheel and Rice, 2013). Current evidence suggests a major role of the structural proteins, with the NS proteins playing a modulating role (Tardif et al., 2002, 2004; Mishima et al., 2010; Von Dem Bussche et al., 2010; Funaoka et al., 2011; Mohl et al., 2012). More work still needs to be done to decipher the mechanisms of UPR induction and the answer will lead to a better understanding of virus-host interaction and may uncover novel mechanisms of UPR sensing in general.

## Author contributions

Shiu-Wan Chan conceived, designed, and wrote the review article.

### Conflict of interest statement

The author declares that the research was conducted in the absence of any commercial or financial relationships that could be construed as a potential conflict of interest.
